# Hydrogen peroxide scavenging is not a virulence determinant in the pathogenesis of *Haemophilus influenzae *type b strain Eagan

**DOI:** 10.1186/1471-2180-6-3

**Published:** 2006-01-23

**Authors:** Bjorn Vergauwen, Mark Herbert, Jozef J Van Beeumen

**Affiliations:** 1 Laboratory of Protein Biochemistry and Protein Engineering, Ghent University, K.L. Ledeganckstraat 35, 9000 Gent, Belgium; 2 Department of Paediatrics, John Radcliffe Hospital, Headington, Oxford OX3 9DU, UK

## Abstract

**Background:**

A potentially lethal flux of hydrogen peroxide (H_2_O_2_) is continuously generated during aerobic metabolism. It follows that aerobic organisms have equipped themselves with specific H_2_O_2 _dismutases and H_2_O_2 _reductases, of which catalase and the alkyl hydroperoxide reductase (AhpR) are the best-studied prokaryotic members. The sequenced *Haemophilus influenzae *Rd genome reveals one catalase, designated HktE, and no AhpR. However, *Haemophilus influenzae *type b strain Eagan (Hib), a causative agent of bacterial sepsis and meningitis in young children, disrupted in its *hktE *gene is not attenuated in virulence, and retains the ability to rapidly scavenge H_2_O_2_. This redundancy in H_2_O_2_-scavenging is accounted for by peroxidatic activity which specifically uses glutathione as the reducing substrate.

**Results:**

We show here that inside acatalasaemic *H. influenzae *all of the residual peroxidatic activity is catalyzed by PGdx, a hybrid peroxiredoxin-glutaredoxin glutathione-dependent peroxidase. *In vitro *kinetic assays on crude *hktE*^- ^*pgdx*^- ^*H. influenzae *Rd extracts revealed the presence of NAD(P)H:peroxide oxidoreductase activity, which, however, appears to be physiologically insignificant because of its low affinity for H_2_O_2 _(*K*_m _= 1.1 mM). Hydroperoxidase-deficient *hktE*^- ^*pgdx*^- ^*H. influenzae *Rd showed a slightly affected aerobic growth phenotype in rich broth, while, in chemically defined medium, growth was completely inhibited by aerobic conditions, unless the medium contained an amino acid/vitamin supplement. To study the role of PGdx in virulence and to assess the requirement of H_2_O_2_-scavenging during the course of infection, both a *pgdx *single mutant and a *pgdx*/*hktE *double mutant of Hib were assayed for virulence in an infant rat model. The ability of both mutant strains to cause bacteremia was unaffected.

**Conclusion:**

Catalase (HktE) and a sole peroxidase (PGdx) account for the majority of scavenging of metabolically generated H_2_O_2 _in the *H. influenzae *cytoplasm. Growth experiments with hydroperoxidase-deficient *hktE*^- ^*pgdx*^- ^*H. influenzae *Rd suggest that the cytotoxicity inflicted by the continuous accumulation of H_2_O_2 _during aerobic growth brings about bacteriostasis rather than bacterial killing. Finally, H_2_O_2_-scavenging is not a determinant of Hib virulence in the infant rat model of infection.

## Background

*Haemophilus influenzae *is a common pathogen among children and immuno-comprised adults with clinical manifestations that are largely type specific. The encapsulated *H. influenzae *serotype b (Hib) usually causes invasive infections, such as meningitis and septicemia [1], whereas the much more common nonencapsulated, or nontypeable, *H. influenzae *is a major cause of otitis media, sinusitis, and pneumonia [2]. *H. influenzae *colonizes the nasopharynx of up to 75% of the population, from where the Hib strains in particular can invade the bloodstream and subsequently pass to the central nervous system. In the course of this pathogenic sequence, the organism moves from sites with high partial oxygen pressure (the nasopharyngeal mucosa; pO_2 _= 100 to 160 mm Hg [3]) to lower oxygenated body compartments (arterial and venous blood and cerebrospinal fluid; pO_2 _= 3 to 100 mm Hg [3]). These latter levels of oxygen, however, are generally sufficient to inflict injury on colonizing bacteria that are strictly anaerobic in nature or that have been deprived of defences against oxygen toxicity [4-7].

Molecular oxygen chemically oxidizes redox centers in all aerobic organisms, generating a flux of hydrogen peroxide (H_2_O_2_) and superoxide radicals (O_2_^•-^) that can potentially damage the cell through chemical modification of cellular building blocks, in the case of DNA leading to an increased and lethal mutation rate [8]. The array of protective measures oxygen-respiring aerobes developed to deal with H_2_O_2_/O_2_^•- ^emphasises the burden that oxidative stress clearly puts on aerobic life. Aerobic organisms generate – or garner from their surroundings – a variety of water- and lipid soluble anti-oxidant compounds. Additionally, virtually all oxygen-respiring organisms contain enzymes that convert O_2_^•- ^and H_2_O_2 _to innocuous products. Moreover, several damage removal/repair enzymes are constitutively synthesized to deal with chemically modified proteins, lipids and DNA. Finally, since O_2_^•-^/H_2_O_2_-levels may vary from time to time – because these levels are the result of a first order chemical reaction with respect to oxygen tension [8], and because such stress can also result from an exogenous source (e.g. bacterial competitors [9] or host inflammatory cells) – organisms are able to adapt to such fluctuating oxidative stresses by inducing the synthesis of antioxidant and damage/repair enzymes. Because of the ubiquity of O_2_^•-^/H_2_O_2_-scavenging enzymes among oxygen-respiring organisms, it follows that scavenging should hold a prominent place among the protective measures against oxygen toxicity.

This prediction was affirmed by studies of superoxide dismutase (SOD)-deficient and hydroperoxidase-deficient mutants of *Escherichia coli*. *sodA*^- ^*sodB*^- ^[10] and *katG*^- ^*katE*^- ^*ahpR*^- ^[11]*E. coli *both suffer elevated rates of oxygen-mediated DNA damage when grown aerobically in rich broth. By characterizing and comparing the H_2_O_2_-sensitivities of acatalasaemic (*katG*^- ^*katE*^-^) *E. coli *and *E. coli *defective in alkyl hydroperoxide reductase (AhpR), Seaver and Imlay [11] found that the H_2_O_2 _dismutase activity of catalase and the H_2_O_2 _peroxidatic activity of AhpR serve redundant, but distinct roles inside the *E. coli *cell. The bipartite alkyl hydroperoxidase system AhpR, composed of a typical 2-cys peroxiredoxin AhpC (the actual peroxidase) and the flavoprotein reductant AhpF [12], are the primary scavengers of endogenous low level H_2_O_2_, while catalase is the more effective scavenger when H_2_O_2_-levels are high and, presumably, when the absence of a carbon source depletes the cell of NAD(P)H necessary for AhpR activity.

Compared to competing bacteria in the upper respiratory tract of humans, *H. influenzae *is generally more sensitive to either oxygen- and H_2_O_2_-mediated cytotoxicity [4,9], which may corroborate with the predicted absence of an AhpR homologue in the sequenced *H. influenzae *Rd genome [13]. Nonetheless, mutating the sole structural gene for catalase, designated *hktE*, does not cause *H. influenzae *type b strain Eagan to grow poorly under aerobic conditions, nor to be reduced in virulence [14]. In fact, acatalasaemic *H. influenzae *Rd compared to its parental strain is not significantly more sensitive to the antimicrobial H_2_O_2_-production of *Streptococcus pneumoniae *[9], implying that catalase does little to protect *H. influenzae *under these conditions.

Additional and efficient hydroperoxidase activity was thus envisioned to be expressed by *H. influenzae*, and recently a candidate structural gene, termed *pgdx*, was cloned and characterized [15]. *pgdx *encodes for the atypical 2-cys peroxiredoxin PGdx, which catalyzes the reduction of both H_2_O_2 _and organic hydroperoxides, specifically by using glutathione – which *H. influenzae *has to garner from its surroundings [16] – as the reducing substrate. Besides its probable role as central H_2_O_2_-scavenger, Murphy *et al*. [17] proposed a function for PGdx in the process of biofilm formation of non-typeable *H. influenzae *during respiratory tract infections, and showed that chronic obstructive pulmonary disease patients persistently colonized with *H. influenzae *can develop antibodies against PGdx.

In this study, a *hktE/pgdx H. influenzae *Rd mutant unable to produce either catalase or PGdx was constructed and evaluated with respect to its sensitivity towards endogenously generated and exogenously supplied H_2_O_2_. These mutations were moved to Hib strain Eagan, the prototypic virulent strain used to assess virulence utilizing the infant rat model, to explore the impact of increased H_2_O_2_-stress on the ability of *H. influenzae *to cause invasive disease. Virulence of the *hktE/pgdx *double mutant was compared with that of the isogenic *pgdx *single mutant strain, and with wild-type virulence established for the Hib strain Eagan parent.

## Results

### Construction of a hydroperoxidase-deficient *H. influenzae *Rd mutant

**Figure 1:**
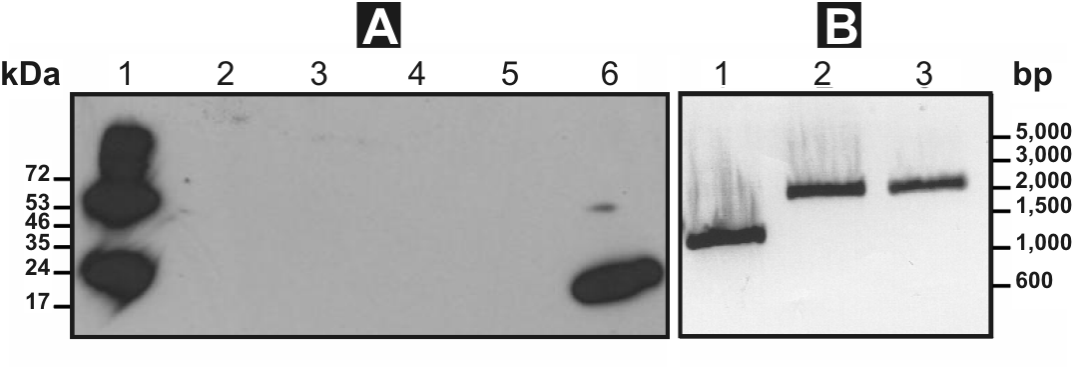
**Western blotting and PCR analysis are consistent with the integration of an ampicillin resistance cassette into the chromosomal *pgdx *gene of the acatalasaemic *H. influenzae *strain AB2593 and the virulent *hktE H. influenzae *type b strain Eagan mutant**. (A) Western blot autoradiogram of PGdx; the preliminary SDS-PAGE experiment was conducted on samples of crude extracts derived from the following cultures; lane 1, *E. coli*, overexpressing PGdx (0.1 μg of total protein); lane 2, *hktE*^- ^*pgdx*^- ^*H. influenzae *Rd (1 μg of total protein); lane 3; *hktE*^- ^*pgdx*^- ^*H. influenzae *Rd (50 μg of total protein); lane 4, *hktE*^- ^*pgdx*^- ^*H. influenzae *type b strain Eagan (1 μg of total protein); lane 5, *hktE*^- ^*pgdx*^- ^*H. influenzae *type b strain Eagan (50 μg of total protein); lane 6, wild-type *H. influenzae *Rd (1 μg of total protein). Monomeric PGdx has a polypeptide mass of 26.6 kDa. Note that the recombinant PGdx protein oxidizes under aerobic conditions to form a disulfide bonded dimer of about 53 kDa. (B) PCR analysis of the *pgdx *gene applied on chromosomal DNA from the following strains; lane 1, wild-type *H. influenzae *Rd; lane 2, *hktE*^- ^*pgdx*^- ^*H. influenzae *Rd; lane 3, *hktE*^- ^*pgdx*^- ^*H. influenzae *type b strain Eagan. The *pgdx *amplicon of 1,071 bp is enlarged by 1,021 bp due to the insertion of the ampicillin resistance cassette.

On the basis of earlier reports [15,18,19], we suspected that the remainder of hydroperoxidase activity in the acatalasaemic *H. influenzae *Rd mutant AB2593 (Rd *hktE*::mini-Tn*10*Cm) could be attributed to the atypical 2-Cys peroxiredoxin PGdx. To explore this hypothesis further, we constructed a derivative of AB2593 in which the *pgdx *gene is insertionally inactivated by an ampicillin resistance cassette (see Methods). One chloramphenicol/ampiccillin resistant colony was isolated and termed *hktE*^- ^*pgdx*^- ^*H. influenzae *Rd from here on. Allelic exchange as a result of a double cross over event and the consequent lack of PGdx expression was confirmed by PCR and Western blotting respectively (Fig. 1).

Because of the probable H_2_O_2_-stress provoked by the disruption of both the *hktE *and *pgdx *genes, *pgdx *disruptants of AB2593 were selected under anaerobic conditions. Unexpectedly, however, *hktE*^- ^*pgdx*^- ^*H. influenzae *Rd colonies were indistinguishable from wild-type colonies when grown aerobically on sBHI plates. On MIc plates, aerobically grown *hktE*^- ^*pgdx*^- ^*H. influenzae *Rd colonies were on the contrary roughly edged and much smaller than their wild-type counterparts (data not shown).

**Figure 2:**
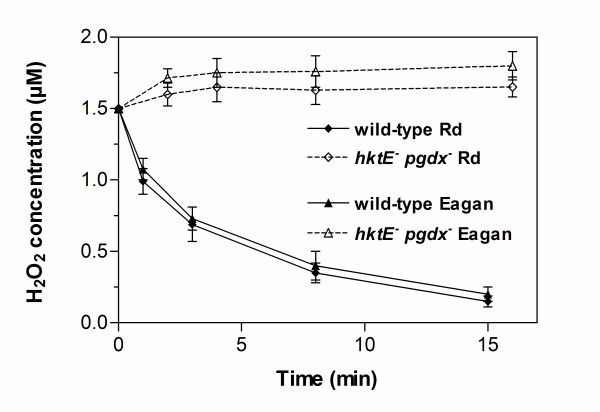
**Both *H. influenzae *Rd and Hib Eagan deficient in both catalase (HktE)and PGdx can be regarded as totally hydroperoxidase-deficient since these double mutants lack the capability to remove low micromolar concentrations of H_2_O_2 _from solution**. To a phosphate-buffered solution of 1.5 μM of H_2_O_2_, 3 × 10^8 ^whole cells of exponentially growing wild-type *H. influenzae *Rd (solid diamonds), *hktE*^- ^*pgdx*^- ^*H. influenzae *Rd (open diamonds), wild-type Hib Eagan (solid triangles), or *hktE*^- ^*pgdx*^- ^Hib Eagan (open triangles) were added, after which the turnover of H_2_O_2 _was followed in function of time (see Methods). Mean values of triplicate experiments (± SEM) are shown.

In order to assess residual hydroperoxidase activity inside *hktE*^- ^*pgdx*^- ^*H. influenzae *Rd cells, the rate of H_2_O_2_-dissipation was measured in a reaction mixture containing 1.5 μM of H_2_O_2 _and 3 × 10^8 ^whole cells of either the wild-type Rd or the *hktE*^- ^*pgdx*^- ^*H. influenzae *Rd strain (Fig. 2). While wild-type cells removed H_2_O_2 _to levels beneath the limit of detection within twenty minutes, no H_2_O_2_-turnover was noticeable in the reaction mixture containing *hktE*^- ^*pgdx*^- ^*H. influenzae *Rd cells, indicating that the double mutant is unable to remove low micromolar concentrations of H_2_O_2 _from solution.

### Low micromolar H_2_O_2_-toxicity is bacteriostatic rather than bacteriocidal to *hktE*^- ^*pgdx*^- ^*H. influenzae *Rd

**Figure 3:**
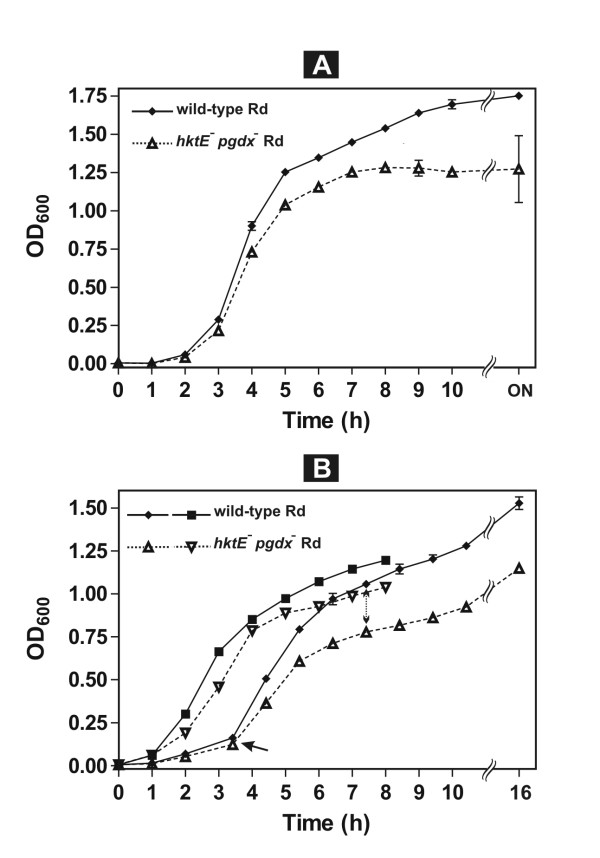
**The scavenging of endogenously generated H_2_O_2 _is not a prerequisite for aerobic growth of *H. influenzae *Rd in rich complex medium**. (A) Aerobic growth of wild-type *H. influenzae *Rd (closed diamonds) and *hktE*^- ^*pgdx*^- ^*H. influenzae *Rd (open triangles) in rich sBHI broth. (B) Anaerobic-to-aerobic shift (indicated by the single arrow) of freshly diluted (1/50) wild-type *H. influenzae *Rd (closed diamonds) and *hktE*^- ^*pgdx*^- ^*H. influenzae *Rd (open triangles) cultures. Of these cultures, 1/50 subcultures in fresh sBHI broth were prepared at the time point indicated by the double arrow, and aerobic growth of these wild-type *H. influenzae *Rd (closed squares) and *hktE*^- ^*pgdx*^- ^*H. influenzae *Rd (open inverted triangles) cultures was subsequently monitored as a measure of their relative fitness at the early stationary phase of growth.

Aerobically grown hydroperoxidase-deficient *katG*^- ^*katE*^- ^*ahpR*^- ^*E. coli *does not survive repeated subculturing in rich Luria-Bertani broth [11]. More precisely, growth rates and final densities are reduced after each dilution, which indicates H_2_O_2_-stress mediated DNA damage [11]. Because of our observation that the *hktE*^- ^*pgdx*^- ^*H. influenzae *Rd double mutant is indistinguishable from its isogenic parent when cultured aerobically on sBHI solid media, we wondered whether the hydroperoxidase-deficient double mutant strain fare as well in aerated liquid medium. Fig. 3A shows the aerobic growth curve of wild-type and *hktE*^- ^*pgdx*^- ^*H. influenzae *Rd cells in sBHI broth. The aerobic growth defect of the hydroperoxidase-negative strain is marginal, though significant, characterized by a slightly lowered doubling time and a reduction in final cell density. More precisely, upon the entry of the early stationary phase of growth, doubling of the *hktE*^- ^*pgdx*^- ^*H. influenzae *Rd culture rapidly stopped. However, no significant decrease in the number of viable cells was apparent when comparing dilutional plating of early stationary phase cultures with overnight cultures (data not shown).

In a previous report, we showed that, because catalase is less efficient in scavenging metabolically-generated H_2_O_2 _than is PGdx, catalase of *H. influenzae *Rd cells that lack functional PGdx (because of the absence of its reductant, i.e. glutathione) is induced about two-fold during routine aerobic growth compared to catalase activity of totally hydroperoxidase-proficient cells [18], while a transient induction of about seven-fold is noticeable after an anaerobic culture was shifted to air [19]. This observation favours the general view that the oxidative stress encountered by microorganisms during an aerobic shift is substantially higher than during routine aerobic growth. So, wild-type and *hktE*^- ^*pgdx*^- ^*H. influenzae *Rd cells were grown anaerobically in sBHI broth to early exponential phase, after which the cultures were shifted to air (Fig. 3B). Hydroperoxidase-deficiency once more did not bring about a fundamental reduction in growth rate in response to the applied oxidative stress, while again lowered the final culture density compared to the isogenic parent. From these anaerobic-to-aerobic shifted cultures, dilutions were prepared at the early stationary phase, and aerobic growth was monitored (Fig. 3B). The resulting growth curves are similar to those obtained from anaerobically pregrown cultures, indicating that the higher level of oxidative stress encountered during the shift to aerobiosis does not cause significant DNA damage to hydroperoxidase-deficient *H. influenzae *Rd grown in rich medium.

**Figure 4:**
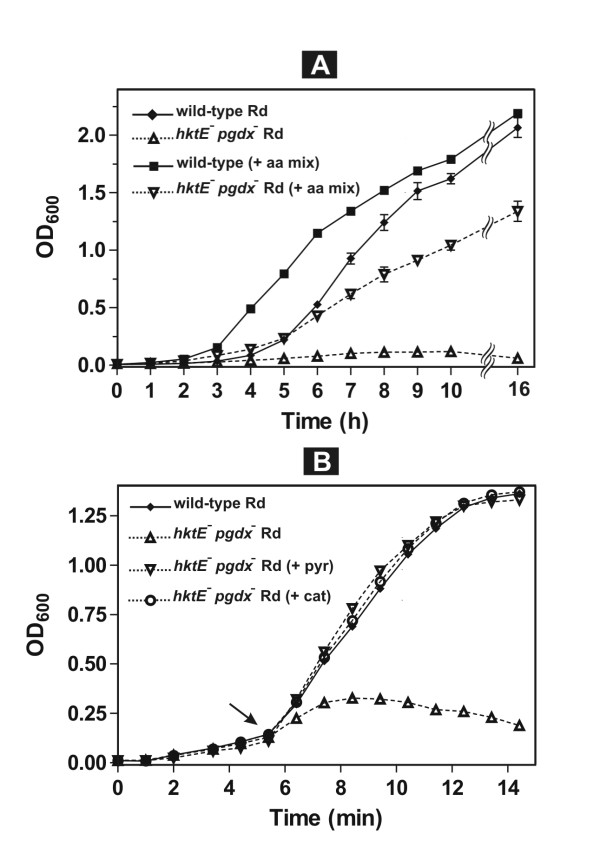
**The scavenging of endogenously generated H_2_O_2 _is a prerequisite for aerobic growth of *H. influenzae *Rd in chemically defined minimal medium**. (A) Aerobic growth of wild-type *H. influenzae *Rd (closed diamonds) and *hktE*^- ^*pgdx*^- ^*H. influenzae *Rd (open triangles) in MIc medium. Aerobic growth of wild-type *H. influenzae *Rd (closed squares) and *hktE*^- ^*pgdx*^- ^*H. influenzae *Rd (open inverted triangles) in MIc medium enriched with a vitamin/amino acid supplement. (B) Anaerobic-to-aerobic shift (indicated by the single arrow) of a freshly diluted (1/50) wild-type *H. influenzae *Rd (closed diamonds) and *hktE*^- ^*pgdx*^- ^*H. influenzae *Rd culture (open triangles) in MIc medium and of a *hktE*^- ^*pgdx*^- ^*H. influenzae *Rd culture in MIc medium either supplemented with pyruvate (inverted open triangles) or catalase (open circles).

On the other hand, growth experiments in chemically defined MIc medium show that under these conditions strain *hktE*^- ^*pgdx*^- ^*H. influenzae *Rd is highly vulnerable to oxidative stress. No growth was observed in fully aerated cultures after dilution (Fig. 4A) or after shifting an anaerobic early exponential phase culture to air (Fig. 4B). Moreover, severe growth retardation was noticeable under the microaerophilic conditions present in a non-shaking candle extinction jar (data not shown). The underlying cause of the encountered oxidative stress is likely to be the accumulation of H_2_O_2_, since the addition of puryvate (a non-enzyme scavenger of H_2_O_2 _[20]) or catalase to aerobically growing *hktE*^- ^*pgdx*^- ^*H. influenzae *Rd cultures resulted in wild-type growth (Fig. 4B).

In 1976, Boehme and coworkers [21] reported that certain amino acid biosynthesis pathways of *E. coli *are extremely vulnerable to oxidative inactivation. To test the hypothesis that aerobically grown *hktE*^- ^*pgdx*^- ^*H. influenzae *Rd cells in MIc medium are not viable because of the inability to synthesize certain amino acids or vitamins, the growth experiments were repeated in MIc medium that was supplemented with all 20 essential amino acids (final concentration of 40 μg/ml), together with the vitamins riboflavin, niacinamide, pyridoxine and thiamine (final concentration of 1 μg/ml) (Fig. 4A). The aerobic growth defect of the *hktE*^- ^*pgdx*^- ^*H. influenzae *Rd strain was largely alleviated by the amino acid/vitamin enrichment of the minimal medium, as inferred from the slightly lower doubling time compared to growth in rich sBHI broth.

**Figure 5:**
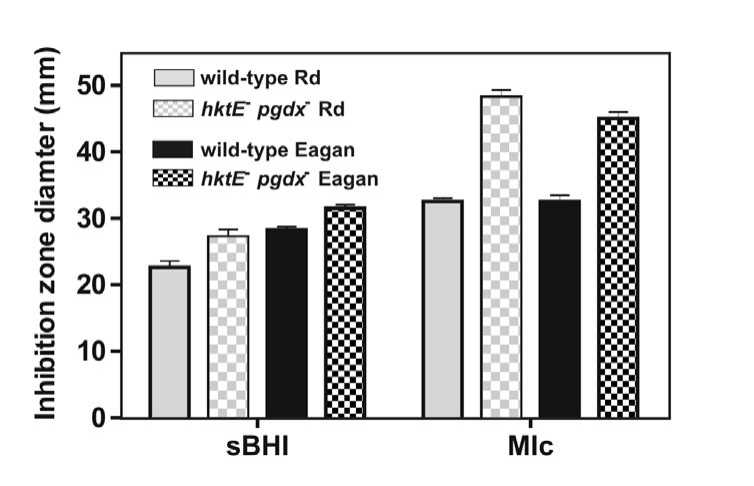
**The *hktE/pgdx *double mutants of *H. influenzae *Rd and Hib Eagan are extremely sensitive to supranormal concentrations of exogenously supplied H_2_O_2_, especially under conditions of low-complex nutrient availability**. Susceptibilities of the wild-type and the *hktE*/*pgdx *double mutant strains of *H. influenzae *Rd and Hib Eagan to H_2_O_2 _were determined by the disk diffusion assay. Disks containing H_2_O_2 _were placed on lawns of exponential-phase pregrown bacteria spread over either sBHI or MIc agar plates and incubated overnight at 37°C under anaerobic conditions. Data represent the mean zone of inhibition (in millimeters) plus the standard error of the mean (SEM) calculated from three independent experiments.

The growth experiments described above are based on the cytotoxicity of low micromolar concentrations of H_2_O_2 _which are inevitably generated during oxygen-respiration [8]. The effect of higher levels of H_2_O_2 _– which e.g. could be the result of the antimicrobial repertoire of bacterial competitors [9] or host phagocytes – on the fitness of the hydroperoxidase-deficient *hktE pgdx *double mutant, was assessed via disk diffusion testing (Fig. 5). Based on inhibition zone diameters, strain *hktE*^- ^*pgdx*^- ^*H. influenzae *Rd assayed on sBHI agar plates was slightly, though significantly more sensitive to the supranormal concentrations of H_2_O_2 _applied on the paper disks compared to the wild-type Rd strain. On the other hand, identical experimental conditions except the use of MIc agar plates as the solidified growth medium, brought about hypersensitivity of the *hktE pgdx *double mutant's condition to high levels of H_2_O_2_. Although more resistant than strain *hktE*^- ^*pgdx*^- ^*H. influenzae *Rd, the wild-type strain was also heavily sensitized to the cytotoxicity of the supranormal levels of H_2_O_2 _when assayed on MIc agar plates.

In summary, respiratory-generated H_2_O_2 _seems to affect growth of hydroperoxidase-deficient *H. influenzae *Rd by blocking the supply of cellular building blocks, resulting in bacteriostasis. It thus appears that *hktE*^- ^*pgdx*^- ^*H. influenzae *Rd prevents the continuously generated stream of H_2_O_2 _being bacteriocidal, either by limiting the ferrous iron-mediated chemical reduction to the extremely harmful hydroxyl radicals (Fenton chemistry [22,23]) or perhaps by having particularly efficient DNA damage repair mechanisms.

### Crude *hktE*^- ^*pgdx*^- ^*H. influenzae *Rd cell extracts contain low-specific NAD(P)H peroxidase activity

**Figure 6:**
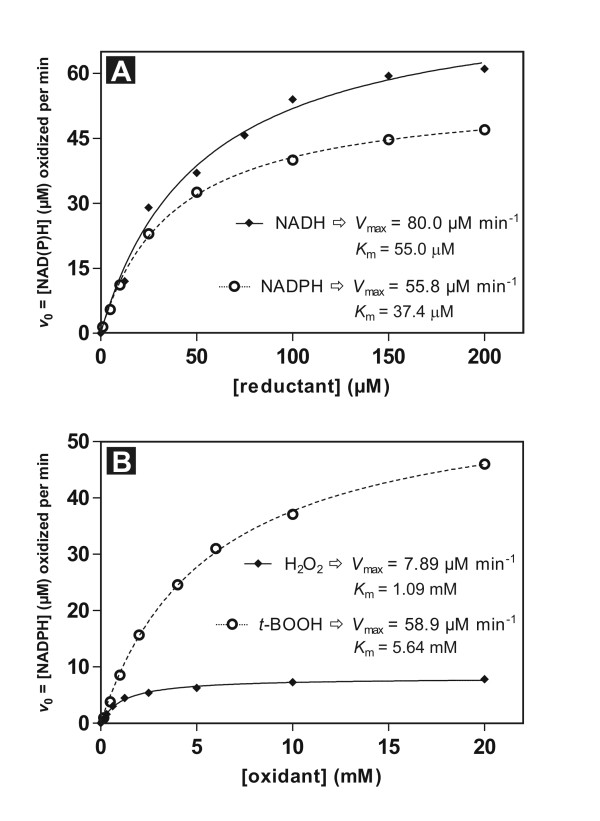
**The NAD(P)H:peroxide oxidoreductase activity found in crude extracts of *hktE*^- ^*pgdx*^- ^*H. influenzae *Rd cultures has physiologically relevant binding affinities for its reducing substrates NADPH and NADH, while being verylow specific with regard to the oxidizing peroxide substrates H_2_O_2 _and *t*-BOOH**. Michaelis-Menten representations of NAD(P)H:peroxide oxidoreductase activity with respect to varying concentrations of (A) reductants and (B) oxidants. Kinetic constants were calculated by fitting the data to the Michaelis-Menten equation using a non-linear curve fit. The amount of total protein used for each kinetic assay was 2.5 mg.

Because PGdx reduces peroxides, while concomitantly oxidizing glutathione, direct spectrophotometric monitoring of *in vitro *PGdx activity is feasible by following the NADPH-dependent reduction of glutathione disulfide catalyzed by the flavoprotein glutathione reductase. By using *t*-butyl hydroperoxide (*t*-BOOH) as the peroxide substrate, the usefulness of this assay to enzymatically confirm the *hktE pgdx *double mutation was limited because of severe background activity, i.e. oxidation of the nicotinamide nucleotide reductant was already apparent in reaction mixtures solely containing NADPH, *t*-BOOH and crude extract. Similar NADPH-dependent peroxidase activity was observed for mixtures containing NADPH, H_2_O_2 _and crude extract. Because this *in vitro *peroxidatic activity conflicts with the previous conclusion that *hktE*^- ^*pgdx*^- ^*H. influenzae *Rd cells are totally devoid of H_2_O_2_-scavenging activity, we determined the kinetic parameters of the NADPH peroxidatic activity using crude extracts (Fig. 6). The saturation curves for the reducing substrates NADPH and NADH (Fig. 6A) yielded comparable specificities, with *K*_m _values resembling their *in vivo *concentrations (*K*_m_-NADPH = 37.4 μM; *K*_m_-NADH = 55.0 μM). On the other hand, the *K*_m _values for the oxidizing substrates *t*-BOOH (*K*_m_-*t*-BOOH = 5.6 mM) and H_2_O_2 _(*K*_m_-H_2_O_2 _= 1.1 mM) are far above physiologically relevant *in vivo *concentrations (Fig. 6B). In fact, the affinity constant for H_2_O_2 _is 3 orders of magnitude higher compared to the *K*_m _value of ~2 μM for PGdx catalyzed H_2_O_2_-reduction [15], likely explaining that this novel NAD(P)H:peroxide oxidoreductase activity is of minor importance when assaying the turnover of low micromolar concentration of H_2_O_2 _by whole *hktE*^- ^*pgdx*^- ^*H. influenzae *Rd cells.

For the NADPH:peroxide oxidoreductase activity to be a determinant factor for the *hktE*^- ^*pgdx*^- ^*H. influenzae *Rd cells remaining as fit under aerobic conditions in rich medium as their isogenic parent, one would expect this peroxidatic activity to be regulated in response to oxidative stress. So, NADPH peroxidatic activity was measured in crude extracts derived from wild-type and *hktE*^- ^*pgdx*^- ^*H. influenzae *Rd cultures using *t*-BOOH as the oxidizing substrate. For the purpose of the present study, monitoring the NADPH-dependent turnover of H_2_O_2 _would be more appropriate, however, the endogenous catalase activity of wild-type cells conflicts with this approach. No induction of NADPH-dependent *t*-BOOH peroxidase activity was apparent. In fact, from the recorded specific activities of 11.8 ± 1.3 nmol/min mg protein and 8.2 ± 2.1 nmol/min mg protein for wild-type and *hktE*^- ^*pgdx*^- ^*H. influenzae *Rd cell extracts respectively, it seems that NADPH:*t*-BOOH oxidoreductase activity is slightly repressed as the result of H_2_O_2_-stress.

### Hydroperoxidase-deficient *H. influenzae *strain Eagan is not attenuated in virulence

To determine the involvement of PGdx in the pathogenic sequence leading to bacteremia and to assess the influence of increased H_2_O_2_-stress on Hib virulence, both a single Hib Eagan mutant disrupted in its *pgdx *gene, and a Hib Eagan *hktE pgdx *double mutant were created by moving these mutations from the genetically modified Rd strains to competence-induced Hib Eagan cells (Fig. 1). Growth of wild-type and *hktE*^- ^*pgdx*^- ^Hib Eagan strains in rich sBHI and chemically defined MIc liquid medium showed similar trends as described for their Rd counterparts (data not shown). Moreover, Fig. 2 shows that the *hktE*^- ^*pgdx*^- ^Hib Eagan double mutant is completely unable to metabolise low μM concentrations of H_2_O_2_, while Fig. 5 shows that the double mutant cells are sensitised to H_2_O_2_-stress due to low-complex nutrient availability. These two phenotypes match those observed for the Rd counterparts and conclusions are to be drawn accordingly. To assess virulence, wild-type Hib Eagan and mutant strains were cultured anaerobically to mid-exponential phase, diluted to ~200 CFU/100 μl, and intraperitoneally inoculated into 5-day-old infant rats. Bacteremia was assessed at 48 hours by culturing a tail vein blood sample anaerobically on sBHI plates containing the appropriate antibiotics. Compared to wild-type Hib Eagan (814 ± 380 CFU/5 μl blood; n = 6), both *pgdx*^- ^(899 ± 248 CFU/5 μl blood; n = 5) and *hktE*^- ^*pgdx*^- ^Hib Eagan (782 ± 433 CFU/5 μl blood; n = 4) were not attenuated in virulence.

## Discussion

A Hib mutant defective in lipoamide dehydrogenase is indistinguishable under anaerobic conditions from its isogenic parent, while showing no growth at all in the presence of air [24]. Because this strain is severely reduced in virulence, it was concluded that Hib requires the ability for aerobic respiration in order to complete its pathogenic sequence leading to invasive disease. It thus follows that Hib strains are subjected to O_2_^•-^/H_2_O_2_-toxicity in the course of bacteremia. Nonetheless, the fitness of our hydroperoxidase-deficient *H. influenzae *Rd mutant is only marginally affected by aerobic conditions in rich sBHI broth, and *hktE*^- ^*pgdx*^- ^Hib Eagan displays a normal ability to produce persistent bacteremia in infant rats. Similar observations are reported for SOD-deficient Hib [4], suggesting that in the infant rat model of infection, neither phagocytic cells and their respiratory bursts, nor the potentially toxic O_2_-levels in the blood, play a major role in limiting Hib virulence. The pathogenic sequence of *Salmonella typhimurium *– another causative agent of bacteremia in humans – to cause infection when injected into mice by the intraperitoneal route, is also indifferent to the presence of either catalase [25], AhpC or OxyR [26] (which regulates transcription of about 30 proteins in response to fluctuating H_2_O_2_-levels [27]), while a severe attenuation in virulence is noticeable for a *Salmonella typhimurium recA *mutant defective in DNA repair [25]. Thus, the ability to repair damaged DNA appears to be more important than the ability to directly inactivate the mediators of oxygen toxicity, O_2_^•- ^and H_2_O_2_, during the course of invasive infection of pathogenic agents that are able to deceive the host inflammatory system.

Acatalasaemic *H. influenzae *Rd does not grow in chemically defined MIc medium, because of an abrogated ability to remove H_2_O_2 _[19]. Because wild-type growth as well as wild-type H_2_O_2_-scavenging activity (of low micromolar levels of H_2_O_2_) is regained simply by adding glutathione to the minimal medium, we envisioned that the remainder of hydroperoxidase activity inside acatalasaemic *H. influenzae *Rd is catalyzed in a glutathione-dependent manner [19]. We hypothesized PGdx to be the most likely candidate to catalyze this activity because of its high specificity for H_2_O_2_-reduction (*k*_cat_/*K*_m _= 5.01 × 10^6 ^s^-1 ^M^-1^) and because completion of its peroxidatic cycle exclusively depends on the presence of glutathione (e.g. thioredoxin can not act as a PGdx reductant) [15]. This hypothesis is confirmed here given that either a *H. influenzae *Rd or a Hib Eagan double mutant that lacks both catalase and PGdx can not catalyze turnover of micromolar amounts of H_2_O_2 _(Fig. 2). Thus, catalase (HktE) and a sole peroxidase (PGdx) account for the majority of H_2_O_2_-scavenging in the *H. influenzae *cytoplasm. The H_2_O_2_-scavenging machinery of *E. coli *also is embodied by catalase (KatG) and a sole peroxidase, in this case AhpC instead of PGdx [11]. Although being clearly dissimilar with regard to the reductive branch of their peroxidatic cycles, the peroxiredoxins AhpC and PGdx can be regarded as being functionally analogous, since i) their kinetic parameters for either the reduction of H_2_O_2 _or *t*-BOOH are very similar [15,28]; ii) they both appear to be of most importance during routine exponential growth (when the H_2_O_2_-concentrations are low and the supply of reductant is high) [11,19]; iii) they both are among the most abundantly expressed proteins [29]; iv) neither PGdx, in case of Hib as reported here, nor AhpC, in case of *S. typhimurium *[26] as well as in case of the acatalasaemic anaerobe *Porphyromonas gingivalis *W83 [30], are required for virulence; and v) they both elicit an immunogenic response when injected into infected models [17,26,31]. The latter observation also shows that virulence and immunity are not necessarily connected, as has also been reported e.g. for the major secretory protein of *Legionella pneumophila*, which results in strong protective immunity, but is apparently nonessential for virulence [32].

On the basis of the established physiologically relevant affinities for its reducing substrates, NADPH (*K*_m _= 37.4 μM) and NADH (*K*_m _= 55.0 μM), the NAD(P)H:peroxide oxidoreductase activity, detected here in crude extracts of both *hktE*^- ^*pgdx*^- ^*H. influenzae *Rd and its wild-type parent, could be of some relevance for the parasite to control its peroxide levels. The affinities, however, for either the simplest peroxide H_2_O_2 _(*K*_m _= 1.1 mM) or the organic peroxide *t*-BOOH (*K*_m _= 5.6 mM) are so low as to question whether these peroxides are the real *in vivo *substrates for the NAD(P)H:peroxide oxidoreductase activity. Moreover, we have shown here in Fig. 2 that *hktE*^- ^*pgdx*^- ^*H. influenzae *Rd as well as *hktE*^- ^*pgdx*^- ^Hib Eagan are totally deprived of H_2_O_2_-scavenging activity, meaning that, not only the so-called NAD(P)H peroxidase, but also other hydroperoxidases potentially expressed by *H. influenzae*, such as the thiol peroxidases Bcp and Tpx [33], are not involved in scavenging endogenously-generated H_2_O_2_. In this respect, the determination of the kinetic parameters for Bcp and Tpx would be of great value to clarify this issue.

A decade ago, Coves *et al*. [34] reported the NAD(P)H: H_2_O_2 _oxidoreductase activity in cell-free *E. coli *extracts; biochemical evidence was provided that such activity was distinct from H_2_O_2_-removal catalyzed by KatG and AhpR. The apparent *K*_m _values of the *E. coli *NAD(P)H peroxidase for its reducing substrates were within 30–40 μM [34], which agree well with our measurements for the *H. influenzae *Rd NAD(P)H peroxidatic activity. Unfortunately, because contaminating catalase activity biased the specific decomposition of H_2_O_2_, no estimation of the binding affinity for H_2_O_2 _of the *E. coli *NAD(P)H peroxidase was provided and no further comparison with the *H. influenzae *activity reported here is currently possible.

Hydroperoxidase-deficient (*katG*^- ^*katE*^- ^*ahpR*^-^) *E. coli *progressively grows slower in rich Luria-Bertani broth and can only grow for two generations in M9 minimal medium supplemented with all 20 amino acids [11]. On the contrary, hydroperoxidase-deficiency (*hktE*^- ^*pgdx*^-^) inflicted in either an Rd or an Eagan background resulted in wild-type growth, generation after generation, in rich sBHI broth, while in chemically defined MIc medium supplemented with all 20 amino acids, a slight aerobic growth defect is manifested as the postponement of nearly wild-type exponential growth to lower-than-wild-type stationary phase cell densities. These results thus imply that endogenously-generated H_2_O_2 _is bacteriocidal (mutagenic) to *E. coli*, while being rather bacteriostatic to *H. influenzae*. This difference in cytotoxic behaviour of H_2_O_2 _can not be attributed to quantitative differences, since we have previously reported that aerobically grown *H. influenzae *Rd cells produce H_2_O_2 _at a similar rate (~12.4 μM/s) as has been established for *E. coli *[11,19]. Two plausible explanations, however, can be put forth to address this issue. First, since H_2_O_2 _by itself is not mutagenic, the rate of formation of mutagenic hydroxyl radicals derived from H_2_O_2 _adjacent to the genomic DNA molecule of *E. coli *may be higher compared to that nearby the *H. influenzae *genome. Secondly, the *H. influenzae *DNA mismatch repair system may be more efficient in repairing its oxidatively damaged DNA than is the *E. coli *counterpart, either on the basis of pure kinetics or because of a difference in sensitivity towards H_2_O_2_-mediated inactivation. In this regard, it is interesting to note that the human DNA mismatch repair system for example is highly sensitive to H_2_O_2_-mediated inactivation, even at noncytotoxic levels of H_2_O_2 _[35]. The present characterization of hydroperoxidase-deficient *H. influenzae *Rd and Hib Eagan suggests that the wild-type strains should be highly robust against H_2_O_2_-stress. How, then, can it be explained that *H. influenzae *is more vulnerable to oxygen toxicity compared to other inhabitants of the human nasopharynx [4,9]? First of all, since we have assessed here cytotoxicity of only one product (H_2_O_2_) of the reaction of oxygen with the cell's redox centers, the possibility remains that growth of *H. influenzae *is directly affected by oxygen toxicity because of intrinsic vulnerability against O_2_^•-^. D'Mello *et al*. [4] indeed reported that growth of *H. influenzae *is highly affected by O_2_^•-^-stress, as evidenced by the absence of growth of a SOD-deficient Hib strain under fully aerated conditions. Secondly, taken into account the highly fastidious nature and the highly condensed genome of *H. influenzae*, O_2_^•-^- and H_2_O_2_-mediated protein inactivation could result in a number of auxotrophies, which can not be relieved under certain culture conditions. In this respect, we consider the basis and diversity of auxotrophies imposed upon *H. influenzae *by either O_2_^•-^- or H_2_O_2_-stress to be important topics for further research.

## Conclusion

By generating mutants of *H. influenzae *Rd and the virulent strain Hib Eagan defective in both HktE and PGdx, we were able to show that these two hydroperoxidases, a catalase and a peroxiredoxin, account for the majority of scavenging of metabolically-generated H_2_O_2_. No other H_2_O_2_-removal activities appear to be of physiological significance. Yet, *in vitro *kinetic assays revealed that *hktE*^- ^*pgdx*^- ^*H. influenzae *Rd still produce NAD(P)H:peroxide oxidoreductase activity. Although this may represent a detoxifying activity, the NAD(P)H:peroxide oxidoreductase appears to be irrelevant for *in vivo *H_2_O_2_-scavenging because of its high *K*_m_-value (1.1 mM) when considering physiological steady-state H_2_O_2_-concentrations (20–150 nM, as estimated for *E. coli *[36,37]). In the absence of hydroperoxidase activity, aerobic growth is fundamentally affected only in case of limiting amino acid/vitamin availability, implying that the continuous stream of H_2_O_2 _generated during *H. influenzae*'s aerobic metabolism affects biosynthetic functions, while, apparently, causing DNA damage which not overkills the cells' DNA repair machinery. In agreement with these *ex vivo *growth studies, in the infant rat model of bacteremia, the double *hktE*^- ^*pgdx*^- ^mutation in the Hib background did not result in attenuated virulence indicating that HktE and PGdx, and more in general H_2_O_2_-scavenging, are not important for virulence in this model of infection.

## Methods

### Materials

Restriction endonucleases were obtained from New England Biolabs (Beverly, MA). DNA purification from gel or solution was carried out using either the Qiaquick DNA Extraction or PCR Purification Kit (Qiagen, Crawley, UK). Ligations were performed using T_4 _DNA ligase (Promega, Madison, WI). Plasmid DNA was prepared by the alkaline lysis method on either a small scale [38] or a 30-ml scale using the Qiagen Plasmid Purification Kit.

### Media

Brain heart infusion broth (BHI) was prepared from a dehydrate (Difco, Becton Dickinson and Company, Franklin Lakes, NJ) and autoclaved. To this medium, a *Haemophilus *test medium supplement (Oxoid, Hampshire, UK), containing V-factor (NAD) and X-factor (hemin), was added according to the manufacturers' instructions to prepare sBHI broth. *H. influenzae *specific minimal medium (MIc medium) was prepared essentially as described by Herriott *et al*. [39]. The MIc medium used in this study contained 50 μM of oxidized glutathione, unless indicated otherwise, and was supplemented with a *Haemophilus *test medium supplement according to the manufacturers' instructions. The amino acid/vitamin supplement used in this study was purchased from Athena Environmental Sciences (Baltimore, MD) and is composed of 19 amino acids (40 μg/ml; no methionine (note that MIc medium already contains methionine)), the vitamins riboflavin, niacinamide, pyridoxine-HCl and thiamine (10 μg/ml each), magnesium sulfate (240 μg/ml), ferrous sulfate heptahydrate (25 μg/ml) and glucose (4 mg/ml). Oxygen-free media were generated using a Coy chamber (Coy Laboratory products, Inc.). To prepare agar plates, 1.8% agar was added to the sBHI or MIc liquid growth media before autoclaving.

### Bacterial strains and growth conditions

*E. coli *TOP10 (Invitrogen, Paisley, UK) was used as host for cloning. *E. coli *strains and clones were cultured at 37°C in Luria-Bertani medium on an orbital shaker rotating at 200 rpm. When appropriate, 100 μg of ampicillin and/or 25 μg of chloramphenicol were added per ml of either solid or liquid *E. coli *culture media.

Wild-type strain *H. influenzae *Rd was purchased from the American Type Culture Collection (Manassas, VA). Strain AB2593 (Rd *hktE*::mini-Tn*10*Cm) was kindly provided by William R. Bishai (Department of Medicine, Devision of Infectious Diseases, Johns Hopkins University School of Medicine). *H. influenzae *type b strain Eagan was kindly provided by A. Wright (Tufts University, Boston).

Cultures were routinely grown at 37°C in a candle extinction jar without shaking. When appropriate, 2 μg of chloramphenicol and/or 6 μg of ampicillin were added per ml of either liquid or solid *H. influenzae *culture media. Growth curves were monitored in the absence of antibiotics and starter cultures were always derived from overnight precultures which were diluted 1:50 to 1:100 to an optical density at 600 nm (OD_600_) of ~0.005. Aerobic growth was monitored under aerobic conditions as described previously [19]. For each growth experiment, three independent experiments were performed with duplicates and the mean of a single representative set of duplicates (± the standard error of the mean (SEM)) is plotted in the figures.

For anaerobic-to-aerobic shift experiments, overnight precultures were diluted 1:50 to 1:100 in oxygen-free growth medium to an OD_600 _of ~0.005 and these subcultures were then grown anaerobically to an OD of ~0.15. Anaerobic cultures were prepared in a Coy chamber under an atmosphere of 85% N_2_-10% H_2_-5% CO_2_. The cultures were subsequently shaken (200 rpm) in atmospheric conditions and OD_600 _readings were recorded at one hour intervals as described previously [19]. In the case of aerobic-shift growth experiments in the presence of non-enzyme or catalase based H_2_O_2 _scavenging, pyruvate (added from a buffered sterile stock solution to a final concentration of 0.75%; Sigma-Aldrich, St. Louis, MO) or bovine catalase (1,000 Units/ml; Sigma-Aldrich) were added to the culture media.

### Construction of bacterial mutants

A *hktE*^- ^*pgdx*^-^*H. influernzae *Rd mutant was constructed by the integrative disruption method using the acatlasaemic strain AB2593 as a recipient. Briefly, plasmid pSG4.0, a TOPO-XL derivative containing 4.0-kb of *H. influenzea *Rd subgenomic DNA encompassing the 726-bp *pgdx *gene (plus 1.4-kb upstream and 2.0-kb downstream) [18], was linearized with *Acc*I, which cuts the plasmid once at base pair 206 of the *pgdx *gene. The PCR amplified and *Acc*I-digested ampicillin resistance cassette of plasmid pACYC177 (forward primer: 5'-CGTCGACTTCTTGAAGACGAAAG-3'; reverse primer: CGTCGACTTACCAATGCTTAATC-3'; *Acc*I sites are underlined) was ligated into the *Acc*I linearized pSG4.0 plasmid. The resulting *pgdx*::Amp^*R *^knockout out plasmid was used as a template for PCR to amplify the entire mutated *pgdx *locus (forward primer: 5'-CGCGGATCCTGCCTGAACTTTCGCGTAATA-3'; reverse primer: 5'-CGCGGATCCTGTTTGATTTGGCGGATGTA-3'). This DNA was then used to transform the competent AB2593 recipient strain using the MIV method [40]. Integration of the linear *pgdx*::Amp^*r *^knockout-out fragment by homologous recombination was selected for on the basis of ampicillin and chloramphenicol resistance, under anaerobic conditions. The disruption was confirmed by PCR and Western analysis (Fig. 1).

An analogous methodology was used in order to construct strain *pgdx*^- ^*H. influenzae *type b Eagan by using the wild-type Hib strain as the recipient. In order to construct strain *hktE*^- ^*pgdx*^- ^*H. influenzae *type b Eagan, chromosomal DNA prepared from AB2593 was used to transform strain *pgdx*^- ^*H. influenzae *type b Eagan by the MIV method [40], and ampicillin/chloramphenicol resistant transformants were selected under anaerobic conditions. Integrative disruption of the *hktE *gene was analyzed by PCR and confirmed by assaying catalase activity in whole cells as described in ref. [41]. The disruption of the *pgdx *gene was once more confirmed by PCR and Western analysis (Fig. 1).

Both adjacent genes of *pgdx *are transcribed in opposite directions [18]. Therefore, we believe that *pgdx *is not part of an operon, and, consequently, insertional inactivation of *pgdx *is expected to cause no polar effects. Moreover, other illegitimate events due to the insertional inactivation process are unlikely because the *pgdx *locus, contained in the interrupted PCR-amplicon used for the integrative disruption strategy, of wild-type and *hktE*^- ^*pgdx*^- ^double mutant strains was entirely PCR amplified and sequenced (data not shown). No substitutions or deletions/insertions apart from the integrated ampicillin resistance cassette have become apparent.

### Disk diffusion

Overnight anaerobically grown precultures were diluted 1:50 to1:100 in either sBHI or MIc medium to an OD_600 _of ~0.005 and then grown microaerobically (non-shaking candle extinction jar) to an OD_600 _of 0.75 (late exponential phase). The following manipulations were then performed inside the Coy chamber. Using a sterile cotton swab, cells were inoculated onto the entire surface of either sBHI or MIc plates. Round sterile filters (5.2-mm diameter) were placed in the center of the plates and spotted with 5 μl of 3% H_2_O_2_. The plates were placed in an anaerobic jar and incubated for two days at 37°C. The diameter of the zone of complete inhibition was recorded in millimeters. The experiments were performed in triplicate; mean values are plotted with error bars representing the SEM.

### H_2_O_2 _scavenging by whole cells

Overnight anaerobically grown precultures were diluted 1:50 to1:100 in sBHI medium to an OD_600 _of ~0.005 and then grown microaerobically (non-shaking candle extinction jar) to an OD_600 _of 0.15. Cells were pelleted in a microcentrifuge, washed twice, and resuspended in 0.5 ml room temperature phosphate-buffered saline to an OD_600 _of 0.15. An equal volume of a 3 μM H_2_O_2 _solution was then added to the cells to initiate the H_2_O_2 _scavenging reaction. At intervals, 200-μl samples were removed, and the reactions were terminated (i.e. the cells were removed) by filtering the reaction mixtures with sterile Millex-GV13 0.22-μm-pore-size filter units (Millipore Products Division, Bedford, Mass.). Residual H_2_O_2 _was then assayed by using the Amplex red hydrogen peroxide/peroxidase assay kit (Molecular Probes, Eugene, Oreg.) as described before [19].

### NAD(P)H:peroxide oxidoreductase activity measurements

The standard assay for NAD(P)H:peroxide oxidoreductase activity is based on the *t*-BOOH-dependent oxidation of NADPH, measured at room temperature by the decrease in absorbance at 340 nm (ε_340_(NADPH) = 6220 cm^-1 ^M^-1^). A Uvikon 943 double beam UV-visible spectrophotometer (Kontron Instruments, Watford, UK) was used for the spectrophotometric measurements. Each assay mixture contained in a final volume of 0.5 ml, 150 μM NADPH, 20 mM *t*-BOOH, 2.5 mg of crude extract (which was cleared of small metabolites by a HiPrep^TM ^26/10 desalting column (Amersham Biosciences, Freiburg, Germany)), and 50 mM Tris-HCl, pH 7.0. In case of affinity measurements of the reducing substrates NADPH and NADH, the fixed concentration of *t*-BOOH used was 20 mM. In case of affinity measurements of the oxidizing substrates *t*-BOOH and H_2_O_2_, the fixed concentration of NADPH used was 400 μM. Protein concentration was determined by the Bradford method [42] with bovine serum albumin as a standard.

### Attenuation studies

Wild-type Hib and mutants were cultured anaerobically (GasPak150 TM in a BBL GasPakPlus generator with a catalyst (Baxter Diagnostics Inc., Medford, MA)) in BHI broth to an OD_490 _of 0.2 to 0.4. The strains were diluted in phosphate-buffered saline (Gibco, Paisley) with 0.1% gelatin (PBSG) to give ~200 colony-forming units (CFU)/100 μl. Counts were confirmed by plating onto BHI agar without antibiotics (data not shown). Approximately 200 CFU of each strain were intraperitoneally inoculated into 5-day-old Sprague-Dawley infant rats. At 48 hours, the rats were bled, and a 5-μl tail vein blood sample was diluted 1:200 in PBSG and cultured anaerobically on BHI agar. Colony counts of recovered Eagan and mutants were compared. Values are means ± standard error of at least four experiments.

## Authors' contributions

BV carried out the molecular genetic work, the enzymological and the physiological studies, and drafted the manuscript. MH carried out the virulence studies and helped to draft the manuscript. JJVB conceived of the study, and participated in its design and coordination. All authors read and approved the final manuscript.
